# Bioactive Compounds as Potential Agents for Sexually Transmitted Diseases Management: A Review to Explore Molecular Mechanisms of Action

**DOI:** 10.3389/fphar.2021.674682

**Published:** 2021-08-24

**Authors:** Javad Sharifi-Rad, Cristina Quispe, Amirhossein Rahavian, Joara Nályda Pereira Carneiro, Janaína Esmeraldo Rocha, Antônio Linkoln Alves Borges Leal, Maria Flaviana Bezerra Morais Braga, Henrique Douglas Melo Coutinho, Anahita Ansari Djafari, Pedro Alarcón-Zapata, Miquel Martorell, Gizem Antika, Tugba Boyunegmez Tumer, Natália Cruz-Martins, Paweł Helon, Paulina Paprocka, Wojciech Koch, Anca Oana Docea, Daniela Calina

**Affiliations:** ^1^ Phytochemistry Research Center, Shahid Beheshti University of Medical Sciences, Tehran, Iran; ^2^ Facultad de Ciencias de La Salud, Universidad Arturo Prat, Iquique, Chile; ^3^ Andrology Research Center, Yazd Reproductive Sciences Institute, Shahid Sadoughi University of Medical Sciences, Yazd, Iran; ^4^ Universidade Regional Do Cariri - URCA. Cel Antônio Luis, Crato-CE, Brazil; ^5^ Department of Urology, Shohada-e-Tajrish Hospital, Shahid Beheshti University of Medical Sciences, Tehran, Iran; ^6^ Clinical Biochemistry and Immunology Department, Faculty of Pharmacy, University of Concepción, Concepción, Chile; ^7^ Facultad de Ciencias de la Salud, Universidad San Sebastián, Concepción, Chile; ^8^ Department of Nutrition and Dietetics, Faculty of Pharmacy, and Centre for Healthy Living, University of Concepción, Concepción, Chile; ^9^ Universidad de Concepción, Unidad de Desarrollo Tecnológico, UDT, Concepción, Chile; ^10^ Department of Molecular Biology and Genetics, Faculty of Arts and Science, Canakkale Onsekiz Mart University, Canakkale, Turkey; ^11^ Faculty of Medicine, University of Porto, Porto, Portugal; ^12^ Institute for Research and Innovation in Health (i3S), University of Porto, Porto, Portugal; ^13^ Laboratory of Neuropsychophysiology, Faculty of Psychology and Education Sciences, University of Porto, Porto, Portugal; ^14^ Branch in Sandomierz, Jan Kochanowski University of Kielce, Sandomierz, Poland; ^15^ Department of Microbiology and Immunology, Institute of Medical Sciences, Collegium Medicum, Jan Kochanowski University in Kielce, Kielce, Poland; ^16^ Chair and Department of Food and Nutrition, Medical University of Lublin, Lublin, Poland; ^17^ Department of Toxicology, University of Medicine and Pharmacy of Craiova, Craiova, Romania; ^18^ Department of Clinical Pharmacy, University of Medicine and Pharmacy of Craiova, Craiova, Romania

**Keywords:** pathogens, natural compounds, mechanisms, pharmacological effects, sexually transmitted diseases (STDs), molecular targets, clinical studies

## Abstract

Sexually transmitted diseases (STDs) are produced by pathogens like bacteria, fungi, parasites, and viruses, and may generate severe health problems such as cancer, ulcers, and even problems in the newborn. This narrative review aims to present updated information about the use of natural bioactive compounds for the prevention and treatment of sexually transmitted infections. A search of the literature was performed using databases and search engines such as PubMed, Scopus, Google Scholar and Science Direct. From the pharmacotherapeutic management point of view, any strategies for prevention should contain medical approaches. The bioactive compounds obtained from natural products have shown biological effects against different microorganisms for the treatment of these diseases. The main results showed antimicrobial, antiprotozoal, antifungal and antiviral effects such as HIV. Also, the molecular mechanisms, signalling pathways and action targets of natural compounds were highlighted, thus justifying bacterial and antifungal inhibition, apoptosis or reduction of viral replication. From the data of our study, we can conclude that natural compounds may be a significant source for adjuvant drugs / complementary therapies in the treatment of STDs. With all these benefits, the future must conduct extensive clinical trials and the development of pharmaceutical nanotechnologies for a greater therapeutic effect.

## Introduction

More than 30 different pathogens are responsible for diagnosing more than one million new cases of sexually transmitted diseases (STDs) daily worldwide, of which *chlamydia*, gonorrhoea, syphilis and trichomoniasis account for the largest share of STDs with 376 patients. millions of cases annually. (Newman et al., 2015; WHO, 2018). STDs are one of the health problems faced especially by developing countries, they usually affect young people of childbearing age, and can be transmitted to newborns during birth, the financial impact for these countries being overwhelming. (Mabey, 2010). STDs have existed for thousands of years, but the most dangerous of these conditions, Acquired Immune Deficiency Syndrome (AIDS), has been recognized since 1984. STDs can have severe complications, such as infertility, cancer, and pregnancy complications; they also had adverse effects on patients' psychosocial health and quality of life. In addition, some of these STDs may facilitate the transmission of HIV, so their control is one of the goals of the World Health Organization. (WHO, 2006; Gottlieb et al., 2014). Species from the *Candida* genus are commensals and can under specific conditions, become opportunistic pathogens.

For the treatment of *Candida albicans*, essential oils (thyme, oregano, cloves) has traditionally been used, with natural antifungal, antibacterial and anti-inflammatory effects (Sharifi-Rad et al., 2021). Over time, aloe vera has shown remarkable effects in eliminating bacterial STDs and decreasing inflammatory processes. A synergistic combination is aloe vera and honey, for an increased antibacterial effect against Trichomonas vaginalis infection and its symptoms (Friedman et al., 2020). Mangosteen (*Garcinia mangostana*) is a fairly rare fruit that grows in Southeast Asia and has been used successfully in the treatment of gonorrhoea infections. The high concentration of vitamin C and antioxidants gives it strong antibacterial effects. Goldenseal is a medicinal plant native to North America. Goldenseal was used by the Cherokee Indians to treat common colds, infections, flu, gastrointestinal and urogenital diseases. Through its bioactive components, the plant has a strong antibacterial, antiviral, antifungal and antiparasitic action. It is thus a recommended alternative remedy for many diseases of a bacterial or viral nature, such as infections with HPV, *C. trachomatis, C. albicans* (Leyte-Lugo et al., 2017). Starting from ethnopharmacological premises, many natural plant extracts/compounds have shown promising activity in combating chronic diseases, according to preliminary studies (Salehi et al., 2021a; Salehi et al., 2021b). Thus, polyphenolic compounds, alkaloids, isoflavonoid glycosides, oils, lipids, saponins and lactone sesquiterpenes have been shown. may have an anti-trichomoniasis effect in cell studies. (Naidoo et al., 2013).

This work aims to present updated information about the use of natural bioactive compounds for the prevention and treatment of STDs. In this review, we illustrate the current landscape of bioactive natural compounds and their potential role in the future therapeutic armamentarium of STDs.

## Review Methodology

We performed research on PubMed, Scopus, Google Scholar and Science Direct using the MeSH terms: “Sexually Transmitted Diseases”, “Bacterial”, “viral”, “HIV infection”, “classification”, “transmission”, “physiopathology”, “diagnosis”, “pharmacology”, “drug therapy”, “therapeutic use”, “plants/chemistry”, “humans”.

Studies that highlighted the targets and molecular mechanisms of action of bioactive compounds with potential therapeutic effects in STDs were included (Heinrich et al., 2020).

### Inclusion Criteria

1. *in vitro* experimental pharmacological studies on cell lines
2. studies with natural compounds and their derivatives in STDs
3. studies that have highlighted the molecular mechanisms of action

Studies with homoeopathic preparations and without natural bioactive compounds and written in a language other than English were excluded. The taxonomy of plant species was made following The Plant List, and the chemical structures were validated using PubChem and SciFinder. All selected articles were analyzed for pharmacological research (*in vitro*/*in vivo* studies, doses and results with mechanisms included), parts of plants used, bioactive compounds identified and their pharmacological properties against sexually transmitted pathogens. The most important mechanisms of action were represented in the figures together with the chemical structures and tables.

## An Overview of the Pathophysiology of Sexually Transmitted Diseases

We can divide STDs into curable and incurable infections. Major curable STDs, including *chlamydia*, gonorrhoea, syphilis, chancroid and trichomoniasis can be treated with appropriate antibiotics, but incurable infections such as genital herpes (HSV), warts (HPV) and HIV usually have long periods of evolution and there is no cure definite treatment for them. (Mabey, 2010; Gottlieb et al., 2014). Using vaccines against HPV and HSV had a great influence in controlling these diseases (Schiller et al., 2012; WHO, 2012; Boda et al., 2018) but there is still a high prevalence of STDs, especially in developing countries (Centers for Disease Control Prevention, 2011; Gottlieb et al., 2014). Pathogens that cause sexually transmitted diseases are classified into several categories: bacterial, viral, fungal, and parasitic (Table 1).

**TABLE 1:** Etiological classification of the most common STDs.

Bacterial	Viral	Protozoal	Fungal	Ectoparasites
*Neisseria gonorrhoeae*	*Herpes simplex virus*	*Trichomonas vaginalis*	*Candida albicans*	*Phthirius pubis*
*Chlamydia trachomatis*	*Human papilloma virus*			*Sarcoptes scabiei*
*Treponema pallidum*	*HIV (AIDS)*			
*Haemophilus ducreyi*	Cytomegalovirus			

### Bacteria

**FIGURE 1:**
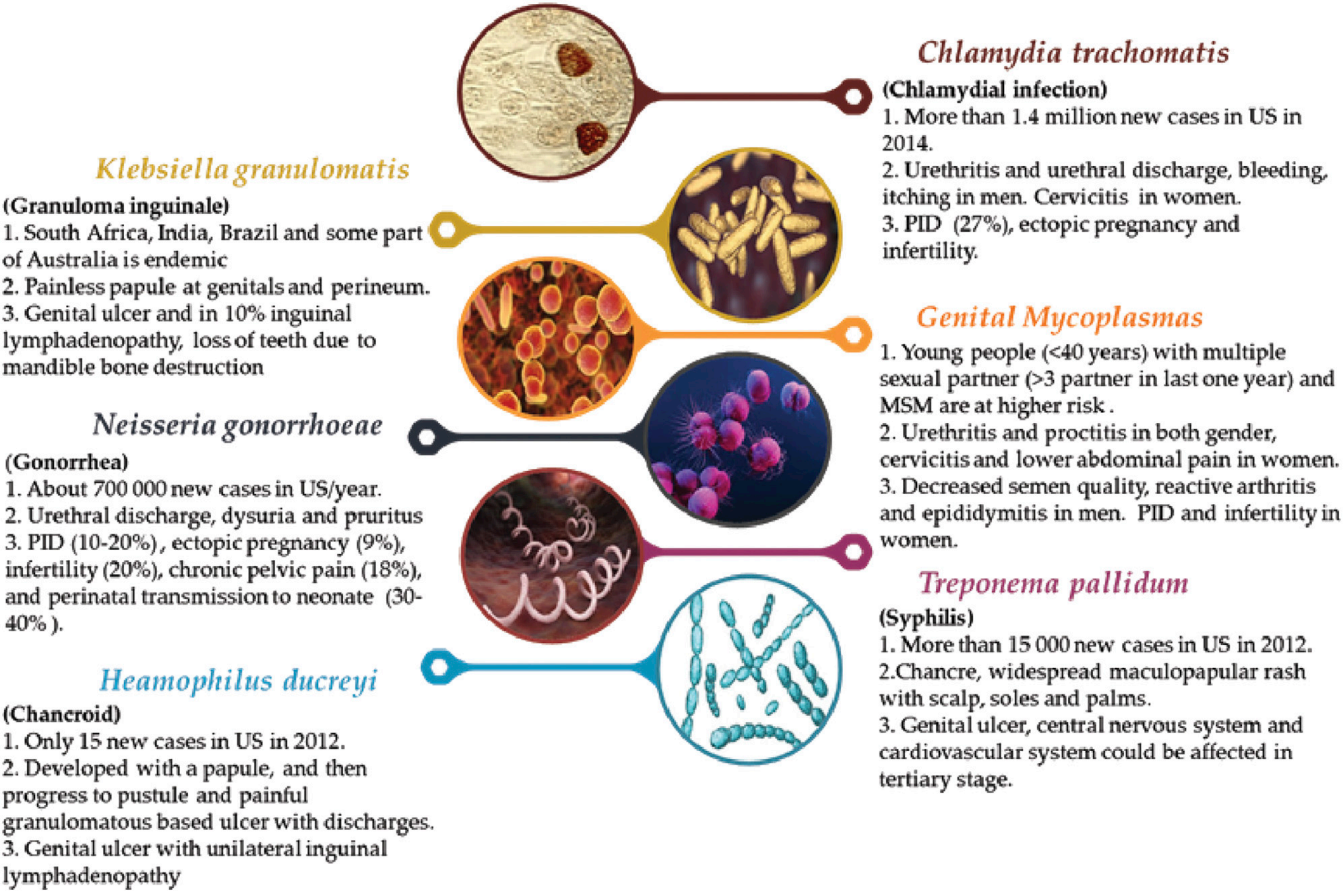
Summary about the major bacteria species that cause STDs (1. frequency for some region, 2. major symptoms, 3. major and possible health effects).

*Neisseria gonorrhoeae* is a Gram-negative bacteria that responsible for gonorrhoea, the second most common STDs in the United States (US) (Figure 1) (Workowski and Berman, 2011). Gonorrhoea causes urethral inflammation and in a rare situation can be disseminated and produce meningitis, dermatitis, endocarditis, arthritis or surgical site infections (Călina et al., 2017). Nowadays performing nucleic acid amplification tests (NAATs) on urine (Geisler et al., 2005) and culture of urethral swab specimens are introduced but NAATs are preferred due to higher sensitivity (Geisler, 2011) (Ungureanu et al., 2017).

Chlamydial infections are the most common bacterial STDs in the world caused by *C trachomatis* (Figure 1). The chlamydial infection affected men and women and transmitted to infants during delivery than about 60–70% of them became symptomatic (conjunctivitis). Traditionally, administration of doxycycline for one week was the choice of treatment but newer studies revealed that a single dose of azithromycin (1 g) is as effective as doxycycline (Lister et al., 1993) and nowadays azithromycin is a suitable choice.

*Haemophilus ducreyi* is a Gram-negative bacteria that caused chancroid (Figure 1) (Lewis, 2003) and painful ulcer with unilateral inguinal lymphadenopathy are manifestations of it. It is difficult to diagnose chancroid by physical exam so some methods were introduced like culture and NAATs but there are not approved by the FDA. Antibiotic therapy is the main treatment and azithromycin, ceftriaxone, ciprofloxacin and erythromycin in different dosage were used (Kemp et al., 2011; Workowski and Berman, 2011).

*Treponema pallidum* is responsible for syphilis (Figure 1) and is transmitted via micro abrasion of skin or mucosa (Ho and Lukehart, 2011). There are different methods for diagnosis of syphilis one of them is identifying the *T. pallidum* from lesion samples under a dark field microscope (Wheeler et al., 2004), Venereal disease Research Laboratory (VDRL), Rapid Plasma Reagin (RPR), Fluorescent Treponemal Antibody absorbed (FTA-ABS) or *T. pallidum* Particle Agglutination (TP-PA) are some serologic test that used (Calonge, 2004). Treatment of syphilis is antibiotic based and penicillin G in different dosages is the choice (Workowski and Berman, 2011).

Mycoplasmas are a large group that only *mycoplasma* and ureaplasma species are pathogenic for a human being (Zdrodowska-Stefanow et al., 2006; Calina et al., 2016). These microorganisms are very small without cell wall and culture of them are very difficult so for detection, NAATs or PCR is choice but they are not available commercially (Palmer et al., 1991; Cazanave et al., 2012). Symptoms of infection included urethritis in both gender, cervicitis and lower abdominal pain in women (Figure 1) (Taylor-Robinson and Jensen, 2011; Jensen et al., 2016) but less than half of patients developed symptoms (Anagrius et al., 2005; Falk et al., 2005). Treatment is antibiotic based and azithromycin is a choice with a cure rate of about 95% (Jensen et al., 2016).

*Klebsiella granulomatis* is a Gram-negative bacterium that caused granuloma inguinale (Donovanosis) (Taheri et al., 2021; Zlatian et al., 2018) (Figure 1). This STD is rare in the US and Europe but in South Africa, India, Brazil and some part of Australia is endemic (Lagergård et al., 2011; Pontari, 2016). About 50 days after exposure, symptoms appeared (O’Farrell, 2002). At the site of infection (genitals and perineum) a painless papule appeared that progressed to ulcer and in 10% inguinal lymphadenopathy may occur. The ulcers are vascular rich so bleed easily (Velho et al., 2008). Lymphadenopathy is uncommon but the inguinal ulcer is common and kissing ulcers on the inguinal fold are characteristic of this infection (Richens, 1991). Other areas that affected are gingivae, lips, mandible, abdomen, arm, leg and bone and oral lesions are more frequent (Galarza, 2000; Richens, 2006) that can cause loss of teeth due to mandible bone destruction (Velho et al., 2008). Diagnosis is based on identifying Donovan bodies under a microscope after Wright, Giemsa or Leishman staining (Richens, 2006) and doxycycline are antibiotics of choice for treatment that should consume till all lesions disappeared (at least three weeks) (Workowski and Berman, 2011).

### Fungus

Candidiasis is the most common fungal infection and *Candida albicans* is the most common species. (Sobel, 2014). There are some risk factors for candidiasis such as pregnancy, diabetes, consumption of antibiotics, contraceptive agents and steroids (Geiger and Foxman, 1996; Sobel, 2016). Diagnosis and treatment are empirical and oral azole derivatives appropriately (85–90%) (Sobel et al., 1995; Blostein et al., 2017). Immunosuppression and uncontrolled diabetes are the most important risk factors of recurrent candidiasis and the causative yeast is usually non-*C. albicans* (*Candida glabrata*) (Pontari, 2016).

### Parasites

*Sarcoptes scabiei* is a mite that caused skin infection in about 300 million people per year (Chosidow, 2006). Mites can be also transmitted by contact with infected clothes and beds (Burkhart et al., 2000) so this parasite can spread between family members. Typical scabies manifestations are scabious burrows and nodules that caused itching, especially at night. Permethrin creams 5%, ivermectin and lindane cream 1% is used for treatment. Lindane should be the last choice because its toxicity affected the central nervous system (CNS) and caused seizures and aplastic anaemia (Chosidow, 2006). This type has psoriasiform and warty lesions on the scalp, palm, soles and neck with less itching. The load of mites on these lesions are very high so this disease is very contagious (Chosidow, 2000; Hay et al., 2012).

*Trichomonas vaginalis* is protozoal that produces trichomoniasis, the most common curable STDs (Johnston and Mabey, 2008). More than 157 million new cases were reported worldwide per year and its incidence is increasing annually (WHO, 2001), its cause is that rate of asymptomatic patients in this disease is high so diagnosis, treatment and cutting transmission chain are difficult. Nowadays using PCR for diagnosis has been studied and the results are impressive and may replace the culture method in future (Wendel et al., 2002; Crucitti et al., 2003; Kissinger et al., 2010; Workowski, 2015). The selective treatment is represented by a single dose of metronidazole (MTZ) or tinidazole is enough but in immunocompromised patients consumption of MTZ for a longer period is recommended (Kissinger et al., 2010). Latex agglutination test and PCR are the other methods that have greater sensitivity than culture but they are not approved yet (Mayta et al., 2000; Crucitti et al., 2003).

Lice are parasites that affect all hairy areas of the body. This disease can affect both sexes of all ages and transmitted directly or indirectly through contact with clothing (Chosidow, 2000). In adults, this disease is classified as STD, but in children, it can be transmitted through individual contact with infected people, so it should not be considered a STD (Chosidow, 2000; Orion et al., 2006). For treatment, permethrin 1% cream is the first choice, also infected clothes must be decontaminated (Orion et al., 2006).

### 3.4. Viruses

HPV is the most common STDs in the world (Chesson et al., 2014) Worldwide prevalence of genital HPV infection was about 630 million (9–13%) and annually 6 million new cases were infected (Centers for Disease Control Prevention, 2007). There is no cure for HPV infection but most HPV infections are self-limited and the median time for clearance of cervical HPV in women and genital HPV in men is 9.4 and 7.5 months accordingly (Lee et al., 2012) and about 90% of low-risk HPV infections regressed within two years so the goals of treatment are: 1) decreasing symptoms and increasing quality of life, 2) reducing viral load to limit transmission and progression (Forcier and Musacchio, 2010). Some treatments are patient-applied like imiquimod 5% cream and podofilox 0.5% solution and some are provider-administered including cryotherapy, surgical therapy and laser therapy for bladder wart (Pontari, 2016). An important issue about HPV is that some types of this virus are the most significant risk factor for cervical cancer and based on this we can categorize HPV infection as low and high risk. Low-risk HPV types included types 11 and 6 that are responsible for 90% of genital warts and low-grade cervical cells changes (Stanley, 2007) and types 16 and 18 were categorized as high risk because associated with invasive and high grade cervical and anal carcinoma (Muñoz et al., 2003). WHO reported about 570,000 new cases of cervical cancer in 2018 (WHO, 2019) and HPV DNA was present in more than 90% of invasive cervical cancer (Chan et al., 2019) furthermore HPV infection caused other malignancies like oropharynx cancers (Forcier and Musacchio, 2010). To reduce the risk of cervical cancer secondary to HPV infection, immunization strategies are the most important goal. (Forcier and Musacchio, 2010; Beachler et al., 2015). Nowadays, there are many types of platforms for obtaining vaccines (Calina et al., 2020a; Calina et al., 2020c), but after clinical studies (Calina et al., 2020b), only three types of vaccines are available for HPV infection:

1) Cervarix: a two valent (2V) vaccine that immunized patients from HPV type 16, 18;
2) Gardasil: a four valent (4V) vaccine that covers HPV type 6,11,16,18;
3) Gardasil 9: a nine-valent (9V) vaccine that targets HPV type 6,11,16,18,31,33,45,52, and 58 (de martel et al., 2017).

It is important to remember that the vaccines are designed for infection prevention not clearing so after tainting, their effect of decrease (Docea et al., 2016; Calina et al., 2020b; Kostoff et al., 2020). Herpes simplex virus (HSV)**:** HSV-1 and HSV-2 are two types of HSV caused by genital herpes and are the most common cause of genital ulcers (Gupta et al., 2007). The worldwide prevalence of genital herpes in 2012 was estimated at 417 million that Africa with 150 million cases had the highest burden (Looker et al., 2015), beside this high prevalence, HSV infection has no definite treatment furthermore many patients do not aware of their disease that increased risk of transmission (Mertz et al., 1992; Gupta et al., 2007) so prevention, diagnosis and treatment of this infection is very important.

HSV-2 infected the genital area only but HSV-1 caused oral herpes and about half of genital herpes (Langenberg et al., 1999). Signs and symptoms of primary genital herpes are various: clusters of erythematous vesicles and papules appear (Corey et al., 1983) after three weeks new lesions appeared and the older ones transformed to pustules and ulcers and finally, these ulcers crusted and healed (Corey et al., 1983). Some patients may develop itching and burning sensation and others may develop malaise, fever and headache, also inguinal and femoral non-tender lymphadenopathy may occur (Corey et al., 1983). Most of the patients have atypical lesions and also differentiation of HSV-1 from HSV-2 based on lesion appearance is impossible so diagnosis based on history and physical examination is not accurate so we need additional testing like viral culture, antigen testing and PCR for diagnosis subgrouping HSV (Centers for Disease Control Prevention, 2002). Herpes virus is incurable and current antiviral agents cannot eradicate the infection, so the aim of treatment is to the alleviation of symptoms, preventing complications like aseptic meningitis and decreasing viral load and risk of transmission (Salehi et al., 2019b; Schneider et al., 2019) Three antiviral drugs including acyclovir, valacyclovir and famciclovir are used that systemic medication is preferred and topical agents are not recommended. Like other STDs the best way for decreasing the prevalence of HSV is prevention.

Human immunodeficiency virus (HIV): is a retrovirus that invades the immune system and T cells, causing decreased CD4 cells and immunity. (Klasse, 2012). HIV-1 and HIV-2 are two subtypes of HIV. HIV-1 is more aggressive and without treatment almost always caused AIDS (Melhuish and Lewthwaite, 2018). Early clinical manifestations of HIV infection occur approximately 4 weeks after exposure and patients may have flu-like symptoms: fever, malaise, arthralgia, myalgia, rash, diarrhoea, pharyngitis and lymphadenopathy (Melhuish and Lewthwaite, 2018).

For HIV diagnosis, serological and virological tests are used; serological tests for HIV antibodies are the most important. If the screening test was positive or suspicious, a confirmatory test should be performed that includes virological tests for antigens, RNA, or an additional antibody test (western blot). The diagnosis of HIV is based on these positive confirmatory tests. (Centers for Disease Control Prevention, 2005). With the introduction of antiretroviral therapy (ART), the epidemiology of HIV has changed. Before ART, the prevalence and incidence and rate of HIV mortality were high; after ART use, the mortality rate and incidence decreased, but the prevalence increased due to the high life expectancy of these patients (HIV/AIDS J.U.N.P.O, 2017). Any strategies for effective prevention should contain structural, behavioural and medical approaches.

*Molluscum Contagiosum* is a skin infection caused by a poxvirus member (genus Molluscipoxvirus) This infection affects both genders and can be transmitted to neonates from pregnant women (Luke and Silverberg, 2010) and is more common in children and transmitted by casual skin-skin contact and towel contact. This virus includes three subtypes, subtype one being the most common. Itching is the most common symptom, but patients can also be asymptomatic. The appearance of characteristic lesions is important for diagnosis, but in the case of atypical lesions or immunocompromised patients, the biopsy of the lesion should be done to rule out malignancy (Van Der Wouden et al., 2017). In the biopsy specimen, Henderson-Patterson bodies or molluscum bodies are important for diagnosis (Eleftheriou et al., 2011). The lesion may spread with shaving and scratching and a bacterial superimposed infection may occur (Desruelles et al., 2013).

If people develop genital *Molluscum contagiosum*, other STDs should be evaluated in the patient (Leung et al., 2017). These lesions are self-limited but it may take 0.5–5 years or longer in immunocompromised patients so treatment is recommended for increasing quality of life (Meza-Romero et al., 2019). Traditional therapies are curettage and cryotherapy but these are painful (Simonart and De Maertelaer, 2008). Nowadays laser therapy is recommended (Binder et al., 2008) also topical therapy with imiquimod, podophyllotoxin, and potassium hydroxide is used. Systemic therapy with cidofovir is useful only in immunocompromised patients.

## Potential Bioactive Compounds Against Sexually Transmitted Diseases Pathogens: Mechanisms and Molecular Targets

### Antibacterial Action

**FIGURE 2:**
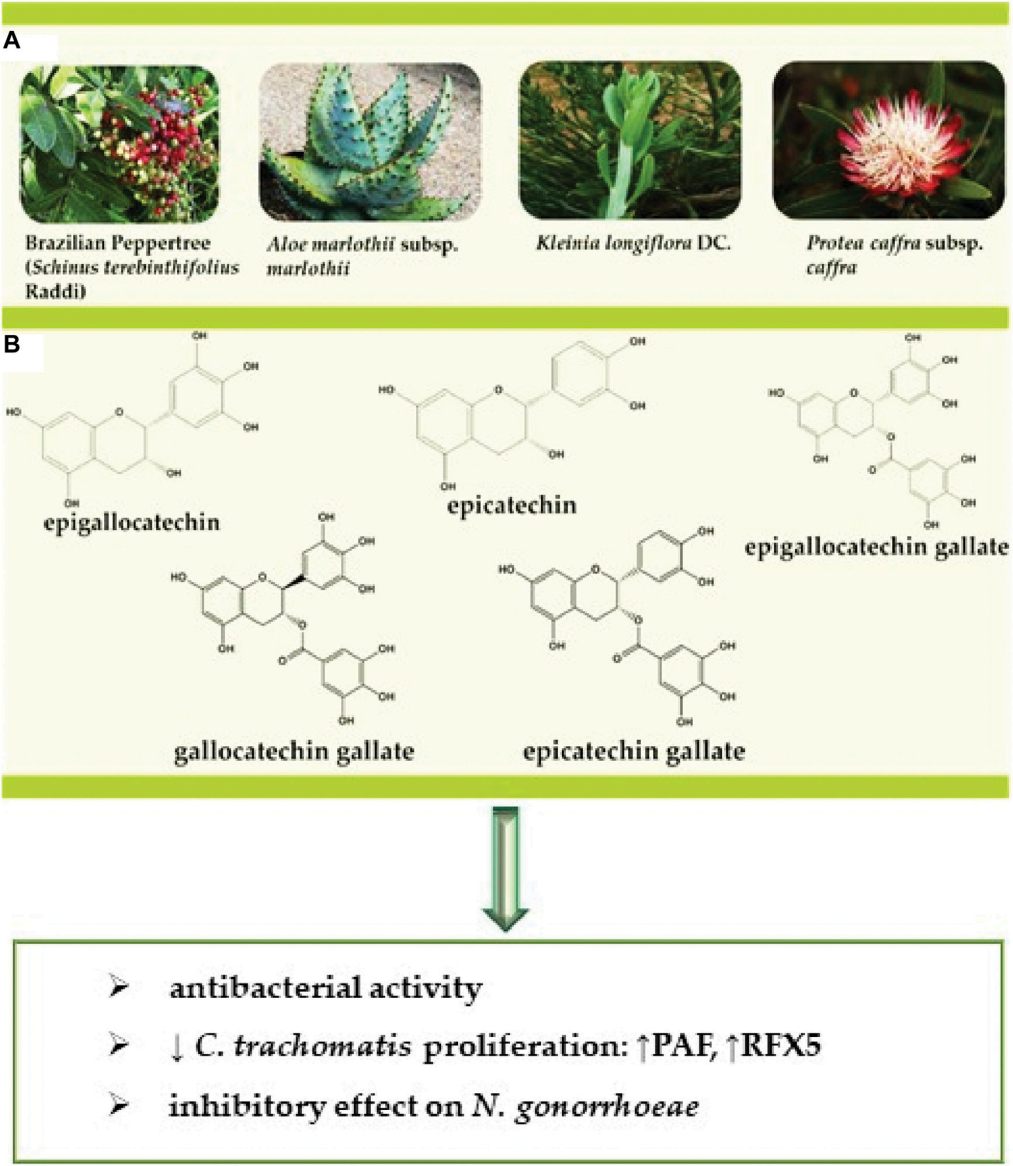
**(A)** Representation of the selected bioactive plant species **(B)** structures of the bioactive compounds against STDs caused by bacteria. Abbreviations and symbols: ↓decrease, chlamydial protease-like activity factor (CPAF), regulatory factor X-5 (RFX5).

Several formulations of commonly used medicinal plant species have exhibited both *in vitro* and *in vivo* anti-chlamydial activity (Figure 2) (Salehi et al., 2019c; Salehi et al., 2019d), including activity in clinical studies, although the specific compounds and mechanisms of action have not yet is determined (Potroz and Cho, 2015; Salehi et al., 2019a).

Polyphenols are natural compounds recognized for their antioxidant, anti-inflammatory, antibacterial, antitumor and anti-atherogenic action (Sharifi-Rad et al., 2020; Salehi et al., 2020). Yamazaki et al. (2003) evaluated the effect of catechin-rich green polyphenol tea extract on *C. trachomatis*-infected human cells, where complete inhibition of *C. trachomatis* occurred from 1.6 mg/ml for serovar D and 0.4 mg/ml for the L2 strain. The polyphenol or Polyphenon 70S comprises epigallocatechin, epicatechin, epigallocatechin gallate, epicatechin gallate, and gallocatechin gallate (Figure 2). Epigallocatechin gallate is the dominant constituent considered to be the major contributor to the observed antimicrobial effects. (Yamazaki et al., 2003). All five catechins showed an *in vitro* inhibitory effect on the proliferation of *C. trachomatis*, and epicatechin was the least toxic (Figure 2). Because the concentration of tea polyphenols required for complete inhibition of *C. trachomatis* is high compared to antibiotics, tea polyphenols are not currently suitable for the treatment of systemic infections. Modifying the structure of catechin may be the solution to reduce the required dose and its toxicity in topical microbicidal pharmaceutical forms.

**FIGURE 3:**
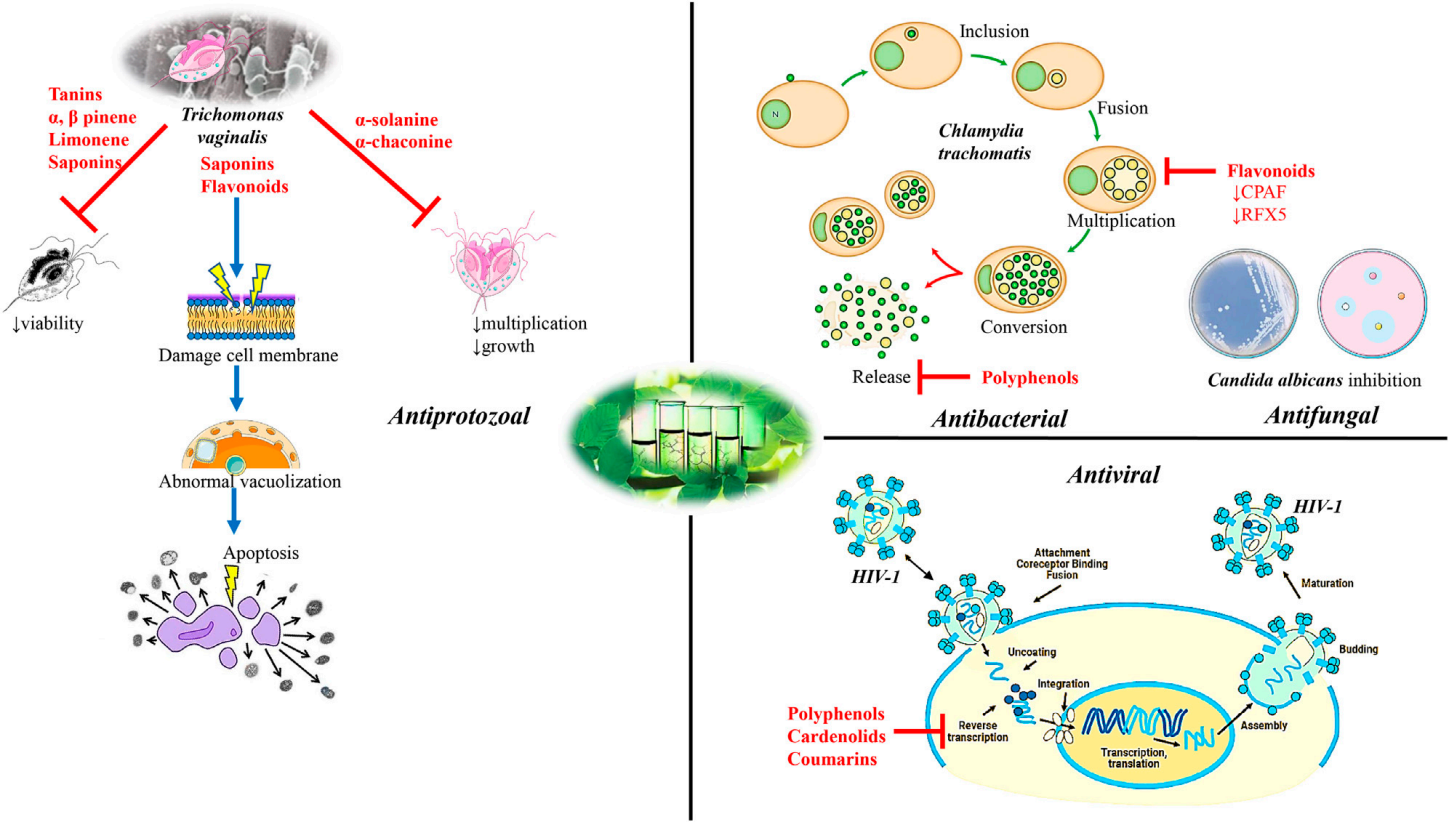
Diagram with the most important molecular mechanisms and targets of natural bioactive compounds as potential alternative agents of STDs. Abbreviations and symbols: ↑increased, ⊥ inhibition, chlamydial protease-like activity factor (CPAF), regulatory factor X-5 (RFX5), human immunodeficiency virus type 1 (HIV-1)*.*

Another study conducted by Hao et al. (2009) showed the flavonoid baicalin derived from *Scutellaria* radix at 0.12, 0.24, 0.48 mg/ml concentrations presented an antibacterial activity against *C. trachomatis* at all tested concentrations. Western blot and RT-PCR assays showed the expression of RFX5 genes during the experiments where the stimulation of CPAF genes protein expression, representative downstream targets of the chlamydial protease-like activity factor (CPAF) in Hep-Cells 2 (Pb0.05). Baicalin presented significant results concerning CPAF protein expression attenuation. From these results, it can be concluded that baicalin can regulate the expression of CPAF and r*egulatory* factor X-5 (RFX5) (Figure 3).

A recent study tested the chloroform extract from three plant species (*Ocimum sanctum* L., *Drynaria quercifolia* (L.) J. Sm., and *Annona squamosa* L. for some of them see Figure 2) for their activity against *N. gonorrhoea* with the disk diffusion method compared with penicillin and ciprofloxacin. Extracts from all three plants presented *Neisseria* growth inhibition at all concentrations (Shokeen et al., 2005).

### Antifungal Action

**FIGURE 4:**
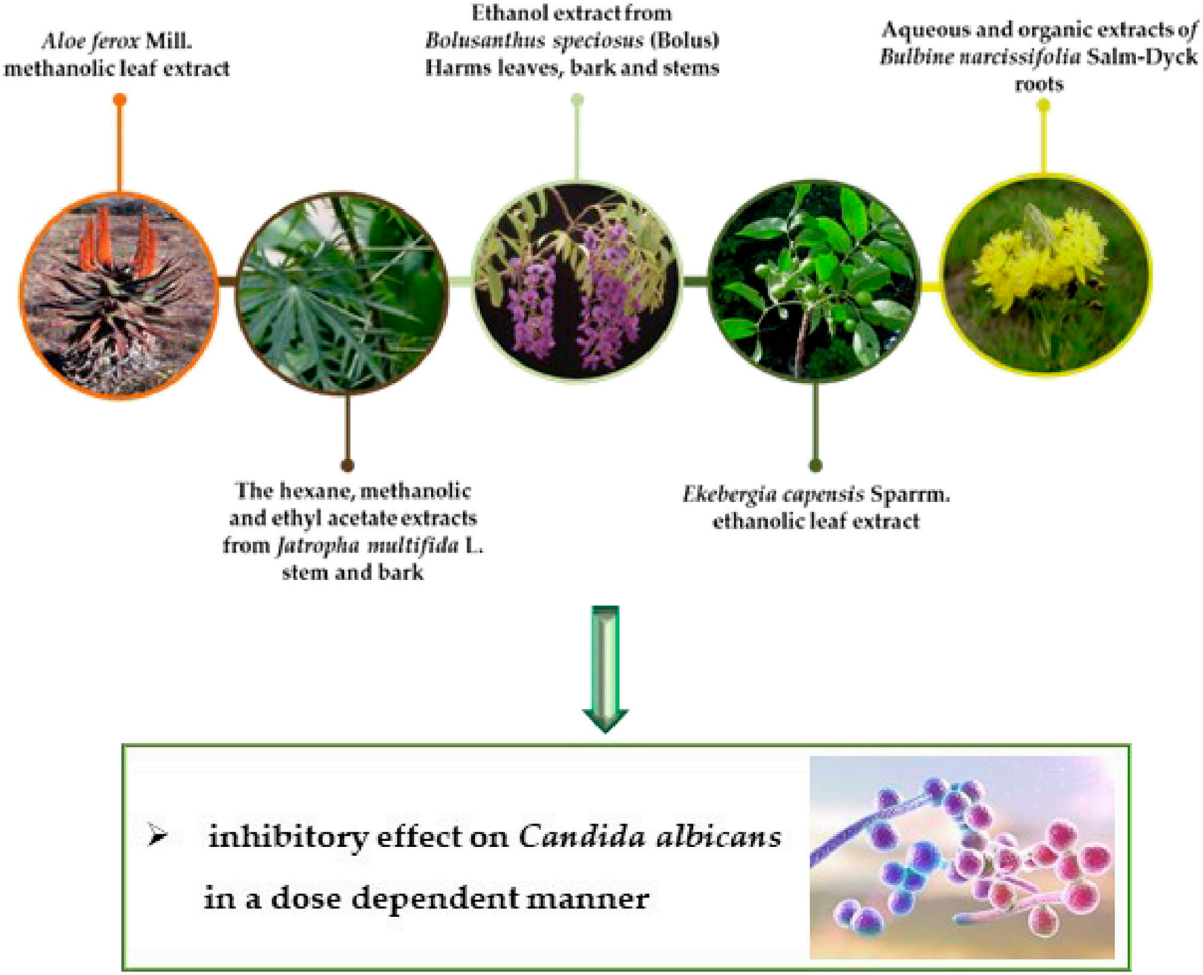
Representation of the selected bioactive plant species against STDs caused by fungus.

Based on ethnopharmacological data, 18 plants were tested against microorganisms associated with urogenital infections/STDs. The dichloromethane: methanol extracts and aqueous extracts presented effects against *C. albicans* (Van Vuuren and Naidoo, 2010). Fractions (50 mg/ml) from 12 plant species were evaluated against *C. albicans*. The best minimum inhibitory concentration (MIC) results were obtained with the dichloromethane and ethanol extract from *Bolusanthus speciosus* (Bolus) Harms leaves (Figure 4, Table 2), bark and stems (0.015–0.193 mg/ml), the ethanolic extracts from *Ekebergia capensis* Sparrm. leaves (0.39 mg/ml), *Pterocarpus angolensis* DC., *Pappea capensis* Eckl. & Zeyh. (0.195 mg/ml), *Ximenia caffra* Sond. (0.78 mg/ml) and *Osyris lanceolata* Hochst. & Steud. roots (0.098 mg/ml). The minimum fungicidal concentration obtained better results with *B. speciosus* (0.012 mg/ml) (Mulaudzi et al., 2011) (Figure 3).

**TABLE 2:** Pharmacological properties of selected plants/natural compounds against STDs pathogens.

Pharmacological activities	Plant used/compounds	Model	Dose	Major findings	Refs.
*Antibacterial effects*					
**Anti-chlamydia**	Catechin-rich green polyphenol tea extract	*C. trachomatis*-infected human cells/*in vitro*	IC_50_ = 1.6 mg/ml	↓ C. *trachomatis* proliferation	Yamazaki et al. (2003)
	*Scutellaria* radix/baicalein	Hep-2 cells infected by *C. trachomatis/in vitro*	IC_50_ = 0.12, 0.24, 0.48 g/ml	↑ RFX5	Hao et al. (2009)
				↓CPAF	
	*Ocimum sanctum* L., *Drynaria quercifolia* L	*N. gonorrhoeae/*disk diffusion method/*in vitro*	IC_50_ = 1,000 μg/L	*↓ N. gonorrhoeae*	Shokeen et al. (2005)
	*Annona squamosa* L./chloroform extract				
***Antifungal effects***					
**Anti-candida**	18 plants/bark, stems, roots dichloromethane:methanol extracts and aqueous extracts	*C. albicans*/*in vitro*	*Bolusanthus* *speciosus*	Antifungal effect	Van Vuuren and Naidoo (2010)
			IC_50_ = 0.015–0.193 mg/ml		
			*Pterocarpus angolensis* DC., *Pappea capensis* eckl. & zeyh		
			IC_50_ = 0.195 mg/ml		
			*Ximenia caffra* sond		
			IC_50_ = 0.78 mg/ml		
			*Osyris lanceolata* hochst. & steud		
			IC_50_ = 0.098 mg/ml		
			*B. speciosus*		
			IC_50_ = 0.012 mg/ml		
	*Sapindus mukorossi* gaertn	*C. albicans*	IC_50_ = 15 mg/ml	Antifungal effect	Talwar et al. (2000)
	*Mentha piperita* L	*C. tropicalis, C. krusei/in vitro*			
	*Azadirachta indica* a				
	13 plant species/aqueous, ethanolic, ethyl acetate extracts	*C. albicans/in vitro*	IC_50_ = 100 mg/ml	Antifungal effect	Buwa and van Staden (2006)
	*Aloe ferox* mill	*C. albicans/*agar diffusion, microdilution	IC_50_ = 20 mg/ml	Antifungal effect	Kambizi and Afolayan (2008)
	*Jatropha multifida* L. stem and bark/hexane, methanolic, ethyl acetate extracts	Methods/*in vitro*			
	19 plant species/organic dichloromethane: Methanol extract, aqueous extracts	*C. albicans/in vitro*	IC_50_ = 0.38–16 mg/ml	Antifungal effect	Jadhav et al. (2015)
	*Acacia karroo* Hayne/roots, *Rhoicissus tridentata* (L.f.) wild & R.B.Drumm/roots	*C. albicans/*broth microdilution method/*in vitro*	IC_50_ = 0.8 mg/ml	Antifungal effect	Mamba et al. (2016)
	*Cassine crocea* (thunb) C.Presl/bark, *Hilliardiella nudicaulis* (DC.)		IC_50_ = 1.6 mg/ml		
	H.Rob./whole plant				
	*P. capensis*	*C. albicans/*broth microdilution method/*in vitro*	IC_50_ = 0.39 mg/ml - 6.25 mg/ml	Antifungal effect	Pendota et al. (2017)
	*Grewia flava* DC., *Jatropha zeyheri* Sond./roots, *Cassia abbrevia* Oliv./leaves	*C. albicans/in vitro*	IC_50_ = 0.20 mg/ml	Antifungal effect	Mongalo et al. (2017)
***Anti- parasites effects***					
**Anti- trichomonas**	*Manilkara rufula* (miq.) H.J.Lam/leaves	*T. vaginalis/in vitro*	IC_50_ = 1.0 mg/ml	Anti-Trichomonas activity	de brum Vieira et al. (2017)
				↓trophozoites viability	
	*Nectandra megapotamica* (spreng.)*/bark, leaves/*essential oil	*T. vaginalis/in vitro*	IC_50_ = 98.7 μg/ml	Anti-Trichomonas activity	Farias et al. (2019)
				↓trophozoites viability	
	*Pistacia lentiscus* L.mastic, *Ocimum basilicum* L./oil	*T. vaginalis/in vitro*	IC_50_ = 15 mg/ml	100% trophozoites growth inhibition damage to the trophozoite membrane system	Ezz eldin and Badawy (2015)
			IC_50_ = 30 μg/ml	↑abnormal cytoplasm vacuolization	
				↑cytoplasm destruction	
	*Rheum ribes* L./flower essential oil/aqueous extract from leaves, flower, stem	*T. vaginalis/in vitro*	IC_50_ = 0.5 mg/ml	Anti-Trichomonas activity	Naemi et al. (2014)
	*Cassine xylocarpa* vent. Leaves, bark, and roots	*T. vaginalis/in vitro*	IC_50_ = 0.46 μg/ml	Anti-Trichomonas activity	Roca-Mézquita et al. (2016)
	*Verbena* sp., *C. xanthocarpa*/aqueous extracts	*T. vaginalis/in vitro*	IC_50_ = 4.0 mg/ml	100% effect against the parasite	Brandelli et al. (2013)
				Complete growth inhibition	
				
	*Hypericum polyanthemum* klotzsch ex reichardt/aqueous extracts	*T. vaginalis* 30,236 (ATCC)*/in vitro*	IC_50_ = 250 μg/ml	Anti-Trichomonas activity	Cargnin et al. (2013)
				Killing 47% of trophozoites	
	*Morinda panamensis* seem roots/anthraquinone	*T. vaginalis/in vitro*	IC_50_ = 1.03 μg/ml	Satisfactory activity against *T. vaginalis*	Cáceres-Castillo et al. (2019)
	*Sapindus/*saponins	*T. vaginalis/in vitro*	Concentration 0,0005%	↓ viability ↓*Trichomonas* trophozoite numbers	Tiwari et al. (2008)
	*Verbascum thapsus* L	*T. vaginalis/in vitro*	IC_50_ = 800 μg/ml	↑apoptosis in trophozoites	Fakhrieh-Kashan et al. (2018)
	*Zingiber officinale* roscoe				
***Antiviral effect***					
**anti-HIV-1 activity**	*Myrothamnus flabellifolia* welw./3,4,5-tri-*O*-galloylquinic acid	M-MLV RT/*in vitro*	IC_50_ = 82 μM	↓HIV-1	Kamng’ona et al. (2011)
				↓RT	
	*Calophyllum brasiliense* cambess/hexanic, acetonic, methanolic extracts	MT2 human lymphocytes	IC_50_ = 0.34 μM/ml	↓HIV-1	Huerta-Reyes et al. (2004a)
		HIV-I RT/*in vitro*	IC_50_ = 0.5 μM/ml	↓RT	
			IC_50_ = 0.66 μM/ml	↓HIV-1 IIIb/LAV ↓replication	
	*Ferula moschata* (H.Reinsch) koso-pol./dry root methanolic extract	H9 lymphocytes/*in vitro*	IC_50_ > 100 mg/ml	↓HIV replication	Zhou et al. (2000)

Abbreviations and symbols: ↑ increased, ↓ decreased, chlamydial protease-like activity factor (CPAF), human immunodeficiency virus type 1 (HIV-1), lymphadenopathy-associated virus (LAV), reverse transcriptase (RT), regulatory factor X-5 (RFX5).

The antifungal effect of two medicinal cream formulations (50%) produced from *Sapindus mukorossi* Gaertn., *Mentha × piperita* L. and *Azadirachta indica* A. Juss herbs have been evaluated to validate their popular use in the treatment of STDs. The formulations presented inhibition halos ranging from 11 to 17 mm against *C. albicans*, *Candida tropicalis*, and *Candida krusei*, compared to fluconazole (15 mg/ml) with halos between 25 and 30 mm (Talwar et al., 2000).

About analysis with extracts, the aqueous, ethanolic, and ethyl acetate extracts (100 mg/ml) from 13 plant species used to treat venereal diseases in Africa were evaluated. The best results with the broth microdilution method against *C. albicans* were obtained with the *Bersama lucens* (Hochst.) Szyszył. bark extract (ethanolic: 0.78 mg/ml) and the *Harpephyllum caffrum* Bernh. bark extracts (aqueous: 1.56 mg/ml; ethanolic: 0.78 mg/ml) compared to amphotericin B (0.195 mg/ml). The results showed an inhibitory effect on these extracts (Buwa and van Staden, 2006).

In another study, Kambizi and Afolayan (2008) obtained *C. albicans* growth inhibition with the *Aloe ferox* Mill (Figure 4). methanolic leaf extract (20 mg/ml) by the agar diffusion method. The hexane, methanolic and ethyl acetate extracts (20 mg/ml) from the *Jatropha multifida* L. stem and bark (Figure 4) were evaluated by agar diffusion and microdilution methods by Aiyelaagbe et al. (2008), where the extracts presented inhibition halos ranging from 11 to 18 mm against *C. albicans*, compared to 20 mm with the Tioconazole.

Zairi et al. (2008) evaluated the antifungal action of dermaseptin-DS4 (peptide derived from frog skin) and its derivatives by microdilution against *C. albicans*. The inhibition against the fungus is dose-dependent inhibiting up to 90% of the population as the concentration was increased.

Naidoo et al. (2013) tested the effects of the organic dichloromethane: methanol extract and the aqueous extract from 19 plant species against *C. albicans*, finding that 11 species presented a potential with the aqueous extract (0.38–16 mg/ml), with *Syzygium cordatum* Hochst. ex Krauss and *Tabernaemontana elegans* Stapf standing out. A total of 16 species presented anti-*Candida* activity (0.5–16 mg/ml), with the best effect produced by *S. cordatum*. Amphotericin B was active at 2.5 μg/ml. The methanolic extract and ethyl acetate fraction (10 mg/ml) extracted from *Terminalia crenulata* Roth. fruits showed antifungal action against *C. albicans* with the agar diffusion method. Inhibition halos ranged from 10 to 15 mm for the extracts and from 36 to 33 mm for fluconazole (25 µg/disc) (Jadhav et al., 2015).

Mamba et al. (2016) observed the antifungal effect of the ethanolic extracts from *Acacia karroo* Hayne (roots), *Rhoicissus tridentata* (L.f.) Wild & R.B.Drumm. (roots), with MICs of 0.8 mg/ml, and *Senna* italic Mill. (roots), *Cassine crocea* (Thunb.) C. Presl (bark) and *Hilliardiella nudicaulis* (DC.) H. Rob. (whole plant), with MICs of 1.6 mg/ml against *C. albicans* with the broth microdilution method more than Ciprofloxacin. The methanolic extract from *Pappea capensis* (100 mg/ml) and the solvent fractions was tested *against C. albicans* by the broth microdilution method, where MIC values ranged from 0.39 mg/ml to 6.25 mg/ml more than amphotericin B (Pendota et al., 2017).

In a South African study, acetone extracts from *Grewia flava* DC. and *Jatropha zeyheri* Sond. roots, as well as *Cassia abbrevia* Oliv. leaves inhibited *C. albicans* growth with a MIC of 0.20 mg/ml. Aqueous extracts from *G. flava* and *Waltheria indica* L. were associated and also presented an effect against *C. albicans.* The amphotericin B control presented an effect at deficient concentrations compared to the extracts (Mongalo et al., 2017).

**FIGURE 5:**
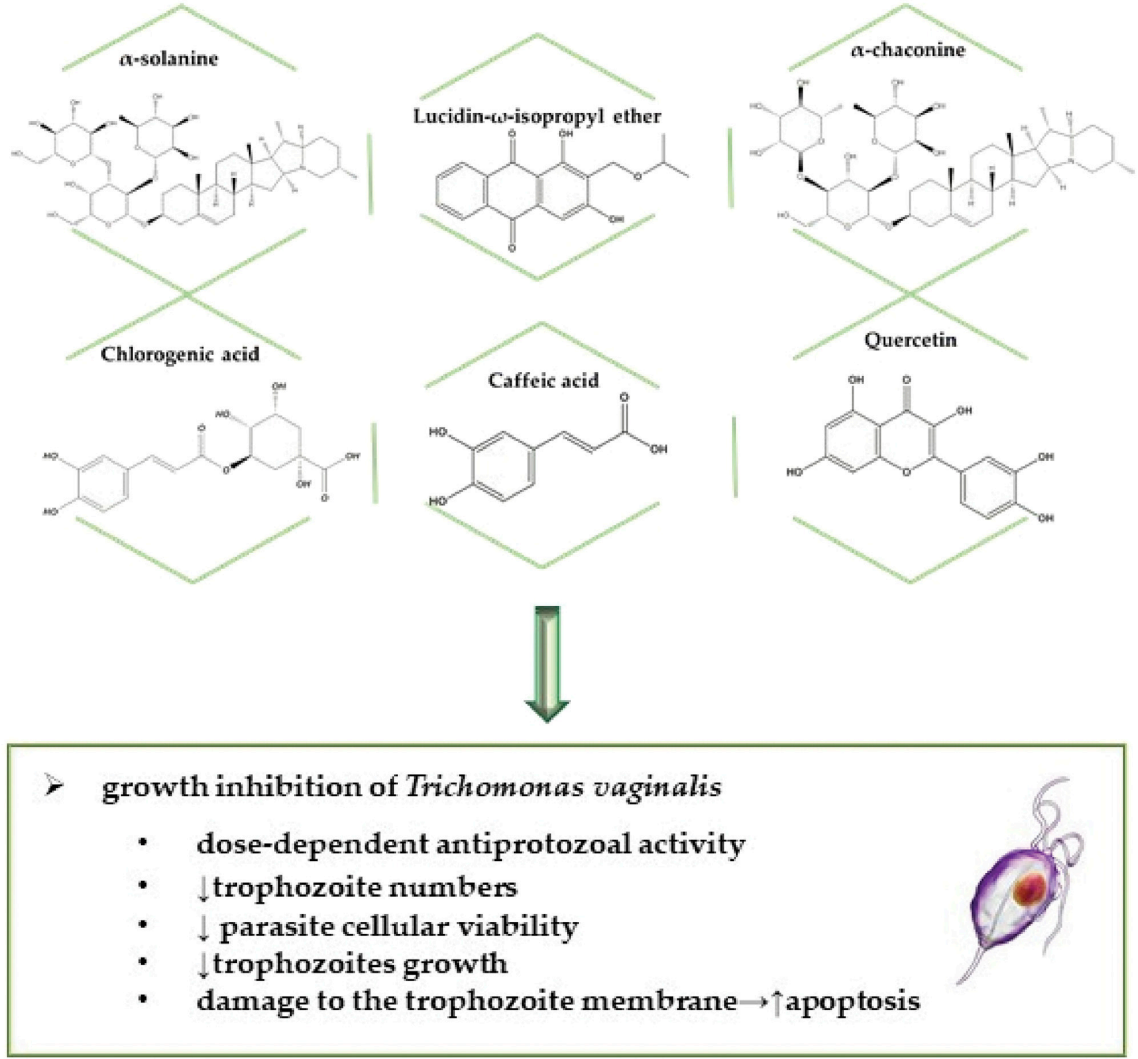
Selected bioactive compounds against STDs caused by parasites, specifically by protozoa.

Seleteng-Kose et al. (2019) evaluated 20 plants against *C. albicans* using their aqueous and organic (dichloromethane: methanol) extracts (leaf and stem roots). Only four plant species, *Bulbine narcissifolia* Salm-Dyck roots (Figure 5) (aqueous and organic extracts), *Helichrysum caespititium* (DC.) Sond. ex Harv. (organic extract), *Phygelius capensis* E. Mey. ex Benth. (aqueous extract) and *Rhamnus prinoides* L'Hér. leaf (organic extract), presented antifungal activity.

### Anti-Parasites Action

In a study conducted by de brum Vieira et al. (2017) evaluated the anti-*T. vaginalis* activity of 7 fractions from *Manilkara rufula* (Miq.) H.J.Lam leaves extracted with acetone and analyze by LC-DAD-MS/MS (liquid chromatography-diode array detector-tandem mass spectrometry), MALDI-MS (matrix-assisted laser desorption/ionization-mass spectrometry), and MALDI-MS/MS. The anti-trichomonas activity and MIC were tested with a 1.0 mg/ml concentration from the fractions or standard compounds (100 mM). The crude fractions reduced *T. vaginalis* viability by over 90%, while all the fractions obtained from ML-A10 reduced trophozoites viability by more than 80%, except the M50 fraction. The M30 and M100 fractions at concentrations from 2.0 to 0.0156 mg/ml were used for the MIC determination. However, none of these concentrations was able to eliminate *T. vaginalis* cellular viability. The M30 fraction consists mainly of flavanols and flavan-3-of derivatives, while the M100 fraction consisted of mainly gallic acid, flavan-3-ol, and flavonols. The author also points out that this is the first time the anti-trichomonas activity of the tannins in question is described. In addition to the anti-*T. vaginalis* activity of the condensed tannins from the M100 *M. rufula* fraction, the author also reports its action against *Tritrichomonas foetus*, thus demonstrating new manners for anti-trichomonas treatment.

Farias et al. (2019) evaluated the essential oil of several species from the genus *Nectandra*. They tested their activity against *T. vaginalis*, in addition to evaluating their antibacterial and biofilm inhibiting effects. The oils were extracted from both the bark and leaves by steam distillation and analyzed by gas chromatography (GC)-MS, with concentrations from 7.8 to 1,000 μg/ml being used for the anti-*T. vaginalis* activity evaluation. The action of the *Nectandra megapotamica* (Spreng.) Mez essential oil was evaluated *in vitro* presented activity against *T. vaginalis* with an IC_50_ of 98.7 μg/ml. The viability of the trophozoites was 1.10 ± 0.69 at the 250 μg/ml concentration and zeroed at 500 μg/ml. The author points out that these are promising results and that α-pinene, β-pinene, and limonene are found in this oil.

Ezz eldin and Badawy (2015) analyzed the *in vitro* effects of *Pistacia lentiscus* L. and *Ocimum basilicum* L. oil against *T. vaginalis* trophozoites. The cultures treated with *P. lentiscus* showed varying degrees of growth inhibition at different time points. However, 100% growth inhibition was observed at a concentration of 15 mg/ml after 24 h of incubation. In cultures treated with *O. basilicum* oil, an inhibition of 100% parasitic growth was observed at a concentration of 30 μg/ml after 24 h of incubation. There was also a 73.1% decrease in trophozoite growth at a concentration of 20 μg/ml after 24 h and complete inhibition of their growth after 48 h. Regarding the transmission electron microscopy (TEM) test, it was shown that both plants cause considerable damage to the cell membranes of trophozoites with abnormal cytoplasm vacuolation, extensive destruction of the cytoplasm, apoptosis with cell death of trophozoites. The author points out the anti - *T. vaginalis* activity of *P. lentiscus* may be attributed to its ability to induce apoptosis and that of *O. basilicum* to active saponin and flavonoid contents found in the oil (Figure 3).

Vazini (2017) evaluated the anti-trichomonas activity of nanoemulsions made from *Mikania cordifolia* (L.f.) Willd*.*. For the test, the drug MTZ was used as a positive control. The *M. cordifolia* nanoemulsion at the 500 ppm concentration is capable of eliminating 85% of trophozoites at 72 h.

Naemi et al. (2014) used the *Rheum ribes* L. flower essential oil in their study, GC-MS analyzed that, and the organic and aqueous extract from the leaves, flower, and stem as well as the 11 fractions obtained. After that, the extracts and fractions were mixed with the parasite. MTZ was used as the control. All fractions presented higher activity at 48 h compared to 24 h. The *R. ribes* flower water fraction presented the highest growth inhibition percentage with the lowest concentration (0.5 mg/ml) after 24 h compared to the positive. The author reports that, according to the results, the aqueous *R. ribes* flower extract may be used for the drug development of anti-trichomoniases drugs.

The dichloromethane and methanol extract from *Cassine xylocarpa* Vent. leaves, bark, and roots were used to evaluate the plant’s activity against several microorganisms, including *T. vaginalis*. The solutions were inoculated with *T. vaginalis* trophozoites followed by incubation, MTZ was used as control. The root bark dichloromethane extract presented an IC_50_ of 0.46 μg/ml against the parasite. The author reports this was the first the extracts as mentioned above were tested; however, only the root bark dichloromethane extract presented activity (Roca-Mézquita et al., 2016).

Ten plants traditionally used by indigenous cultures were tested for their *in vitro* anti-*T. vaginalis* activity including *Aloe arborescens* Mill. leaves, *Bidens pilosa* L. aerial parts, *Rhipsalis baccifera* (J.S.Muell.) Stearn aerial parts, *Luehea divaricata* Mart. barks, *Trichilia* sp. roots, *Campomanesia xanthocarpa* (Mart.) O. Berg leaves, *Coix lacryma-jobi* L. leaves., *Citrus limon* (L.) Osbeck leaves, *Citrus reticulata* Blanco leaves and *Verbena* sp. leaves. From the tested extracts, the *Verbena* sp. and *C. xanthocarpa* aqueous extracts presented the highest activity against *T. vaginalis* with MIC values of 4.0 mg/ml, with *Verbena* sp. presenting 100% effect against the parasite and *C. xanthocarpa* up to 96% against all isolates. MTZ was used as the control. Kinetic growth assays showed the extracts promoted complete growth inhibition after 4 h of incubation (Brandelli et al., 2013).

Cargnin et al. (2013) evaluated the activity of the *Hypericum polyanthemum* Klotzsch ex Reichardt extract and the chemical compounds isolated and purified from it (benzopyrans HP1, HP2, HP3, and the phloroglucinol derivative uliginosin B) against *T. vaginalis* trophozoites. Two *T. vaginalis* isolates were used in the assay, 30,236 (ATCC), which is sensitive to MTZ and the MTZ-resistant isolate TV-LACM2. The following were used for the controls: 8.0 μM MTZ for the sensitive *T. vaginalis* isolate (30,236) and 289 μM MTZ for the resistant isolate (TV -LACM2). The antiprotozoal activity of all isolated compounds was dose-dependent, with the phloroglucinol derivative being the most active. The benzopyran HP1 obtained a selective index value of 73.95, proving to be the most promising compound with significant antiprotozoal activity and selectivity. HP1 presented activity against a resistant clinical isolate (TV-LACM2), killing 47% of trophozoites at the highest concentration (250 μg/ml). Additionally, HP1 with 8.0 μM of MTZ showed a synergistic effect, improved the action of MTZ.

An anthraquinone derived from *Morinda panamensis* Seem. roots were isolated and purified, having its action against *T. vaginalis* evaluated. MTZ 6.0 μM was used as the control. The lucidin-ω-isopropyl ether (Figure 5) activity was examined in axenic trophozoites by IC_50_. Following 24 h of exposure, the substance presented a satisfactory activity against *T. vaginalis* when incubated with varying concentrations (0–20 μg/ml). The inhibition (∼91%) observed with the 20 μg/ml lucidin-ω-isopropyl ether concentration was comparable to the inhibition (∼95%) observed after treatment with the control. The author reports that although anthraquinone presented anti-*T. vaginalis* activity, additional mode of action studies is needed to elucidate the antitrichomonal mechanism of action. (Cáceres-Castillo et al., 2019).

Tiwari et al. (2008) evaluated the anti-*T. vaginalis* activity of *Sapindus* saponins, a component of the plant-based contraceptive Consap, where the *Sapindus* saponins purified sample and MTZ was used. *T. vaginalis* susceptibility was tested being incubated in the presence of serially diluted MTZ (1–12 mM) and *Sapindus* saponins. No growth was observed after 24 and 48 h of incubation at 0.005% saponin concentration. *Sapindus* saponins reduced viability and *Trichomonas* trophozoite numbers at 0.001 and 0.0025% concentrations without live parasites being observed at the 0.005% saponin concentration (MIC) at 12 h.

Frasson et al. (2012) tested 23 different plant species against *T. vaginalis* isolates, including (ATCC) 30,236 and 30,238, and four fresh clinical isolates, TV-LACH1, TV-LACH2, TV-LACM1 and TV- LACM2, where the activity of 44 aqueous extracts from *Caatinga* plants was screened against *T. vaginalis* trophozoites (ATCC 30236). Only the extract that reduced parasite viability by at least 50%, in this case, the aqueous extract, was used in the next experiments. Species belonging to the Polygalaceae family were effective at significantly reducing trophozoite viability, where the *Polygala decumbens* A.W. Benn. root aqueous extract abolished trophozoite viability. The extract was capable of completely impairing parasite growth after the first hours of incubation.

Several substances were evaluated to analyze their anti-*T. vaginalis* activity, including caffeic acid, chlorogenic acid, α-chaconine, solanidine α-solanine and quercetin from frozen potato skin preparations. Parasite growth inhibition by the potato skin compounds was screened against the *T. vaginalis* G3 strain for growth inhibition. The α-solanine inhibitory activity was several times greater than α-chaconine. Potato phenolic compounds also inhibited the growth of the aforementioned strain however, inhibition was much smaller compared to the two glycoalcaldes described above. The potato skin inhibitory activity demonstrated that all skins were active in the assay. The potato samples with the highest activity were the thickest skin varieties and thin skin peels. The study showed the glycoalkaloid potato α-chaconine and α-solanine, the phenolic potato compound chlorogenic acid, caffeic acid and quercetin (Figure 5), as well as potato skins prepared from commercial potatoes, presented antiprotozoal activity against pathogenic *Trichomonas* strains that infect humans, where two Russet samples were the most active (Friedman et al., 2018).

*Verbascum thapsus* L. and *Zingiber officinale* Roscoe plant extracts were analyzed concerning their action against *T. vaginalis*. Trophozoites were cultured with different concentrations of the *V. thapsus* and *Z. officinale* ethanolic extracts and MTZ combinations (concentrations from 25 to 800 μg/ml at 12, 24 and 48 h). The toxicity of the *V. thapsus* and *Z. officinale* ethanolic extract combination at the highest concentration (800 μg/ml) at 48 h was observed to be 1.98% in macrophage cells. At some concentrations the induction of apoptosis in trophozoites was observed (Fakhrieh-Kashan et al., 2018) (Figure 3).

A systematic review published Ziaei Hezarjaribi et al. (2019) by of several plants that possess anti-*T. vaginalis* activity indicated that *Mentha longifolia* (L.) L. is one of the most effective medicinal plants against *T. vaginalis* (31). Additionally, *Eucalyptus camaldulensis* Dehn. presented significant inhibitory effects against *T. vaginalis* and may be used as a safer and more effective alternative to chemical agents for the treatment of this infection in the future.

### Anti-Viral Effects

**FIGURE 6:**
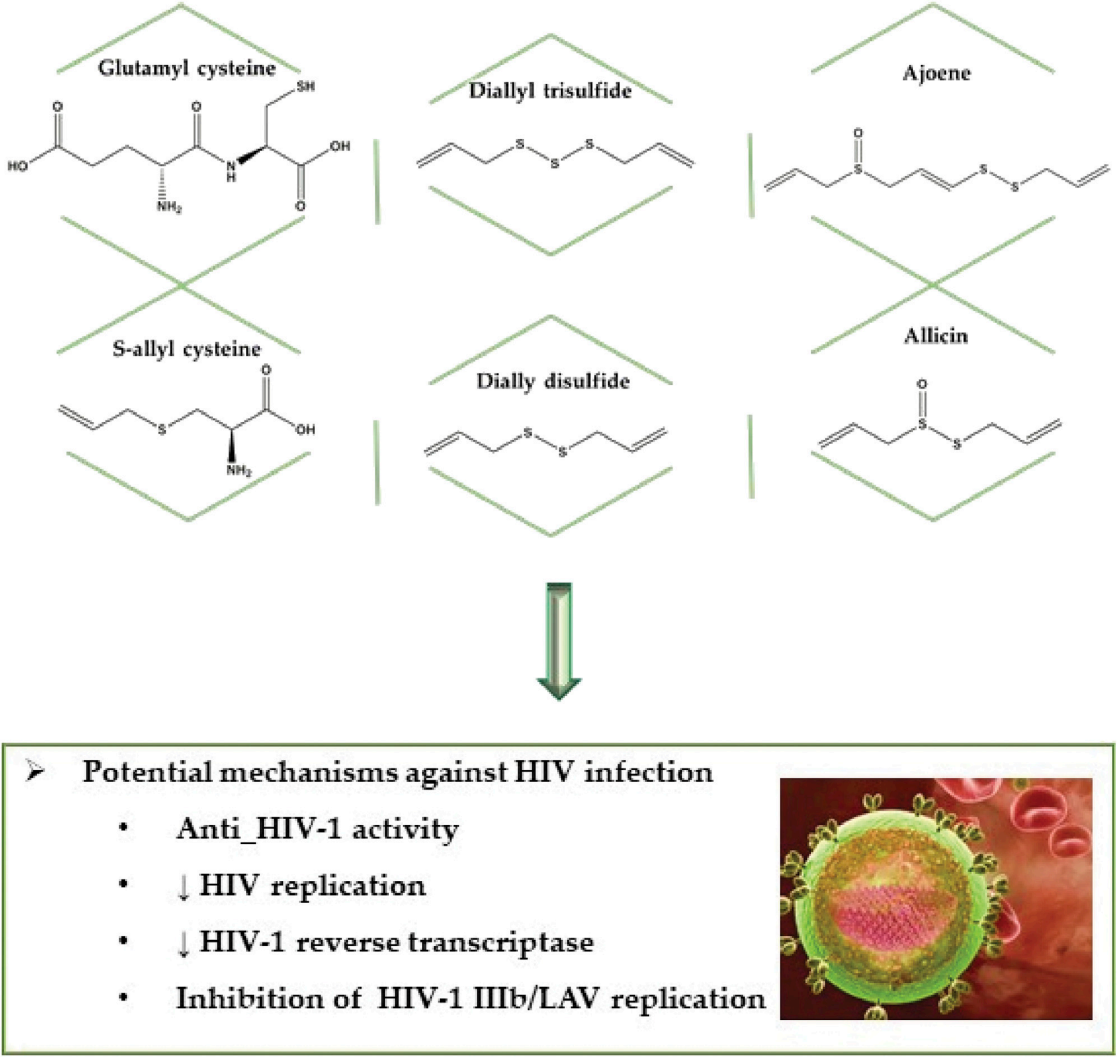
Bioactive compounds against human immunodeficiency virus type 1 (HIV-1) virus.

Gokalp (2018) evaluated the inhibition of some garlic-derived compounds on HIV-1 as well as its action on the most commonly used drug (saquinavir) to treat the disease. Garlic derivatives that inhibit HIV-1 activity are, in ascending order, glutamyl cysteine > ajoene > diallyl trisulfide (DATS)>allin > s-allyl cysteine > dially disulfide > allicin > diallyl sulfide (Figure 6, Table 2).

As for the garlic compound combined with the drug inhibitory activity order, glutamyl cysteine > ajoene > DATS > allin > s-allyl cysteine > allicin > dially disulfide > diallyl sulfide were found to be in ascending order. The author also reports the importance of the anti-HIV-1 activity of garlic compounds, especially ajoene, however, also draws attention to the interaction between these compounds and the saquinavir drug.

Kamng'ona et al. (2011) fractionated the polyphenol-rich extract from the *Myrothamnus flabellifolia* Welw. resurrection plant and isolated the most effective inhibiting fraction in his study. The compound 3,4,5-tri-*O*-galloylquinic acid was identified and analyzed to see if it inhibited the HIV-1 virus Reverse transcriptase (RT) enzyme, where the compound used in the study was extracted from dried *M. flabellifolia* leaves. The HIV-1 RT inhibitory activity of the polyphenol-rich extract and the inhibition kinetics of the 3,4,5-tri-O-galloylquinic acid compound was also evaluated. The results showed an exponential response of HIV-1 RT inhibition over a concentration range of up to 82 μM with an estimated IC_50_ of 34 μM. The effect of nevirapine (drug) on HIV-1 RT activity was also analyzed. The results showed the addition of nevirapine to the 1 mM RT assay caused twice the inhibition observed, however, the highest concentration was insufficient to reduce RT activity by 50%. Thus, the results obtained suggest that 3,4,5-tri-O-galloylquinic acid and its associated compounds may be useful as components of natural antiviral therapy.

The HIV-I RT *in vitro* inhibitory capacity of 21 species from the Clusiaceae family was analyzed, as well as its toxicity in human lymphocytes and inhibition of HIV-1 replication.

For the *Calophyllum brasiliense* Cambess. species organic extracts were also prepared. The antiviral activity assay was performed in three steps, with first the inhibition of HIV-I RT being evaluated and those showing inhibition greater than 70% were used in the next assay addressing cytotoxicity in human cells and the extracts considered as non-toxic were used to determine HIV-1 IIIb/LAV replication inhibition. The drug nevirapine was used as a positive control. Five species (23.8%) showed a high anti-HIV-1 RT (≥70% inhibition) activity, while 7 species (33.3%) were moderately active (50–70% inhibition) and 9 species showed less than 50% inhibition, where the most active extracts were from the same species, *C. brasiliense*. *Clusia massoniana* Lundell and *Vismia baccifera* (L.) Planch. & Triana extracts presented a 72.9% enzyme inhibition*,* while *Clusia guatemalensis* Hemsl. and *Vismia camparaguey* Sprague & L. Riley showed 70.8% inhibition. The toxicity assay was performed with 9 extracts and from these only 5 did not show toxicity: *C. brasiliense* (hexane, methanol and diclorometahne-methanol), *V. baccifera* and *Clusia quadrangula* Bartlett. For evaluation of the HIV-1 IIIb/LAV replication inhibition, the *C. brasiliense* hexane extract inhibited HIV-1 IIIb/LAV replication by 74.5%, while the methanol extract *C. brasiliense*, *V. baccifera* and *C. quadrangula* extracts exhibited less than 52% inhibition. The data obtained in this study suggests the *C. brasiliense* hexane extract possesses anti-HIV activities. Recently the authors identified the existence of 2°*C. brasiliense* chemotypes in Mexico. The first one is included in this study and presented a high inhibitory value for the hexane, acetone and methanol extracts in the HIV-1 RT assay, suggesting these may possess HIV-1 inhibitory substances (Huerta-Reyes et al., 2004b). Using the same previously reported methodology, the authors fractionated and evaluated the hexanoic, acetonic and methanolic extracts from *C. brasiliense* leaves, where the plant extracts were dried at room temperature. Most of the fractions obtained from the *C. brasiliense* hexane leaf extract presented low HIV-1 RT inhibition, however, fractions 18 and 19 showed high inhibition (over 70%) with these fractions also not showing toxicity to MT2 human lymphocytes as well as strongly inhibiting IIIb/LAV replication. Compounds 6 (IC_50_ 0.34 μM/ml), 7 (IC_50_ 0.5 μM/ml) and 9 (IC_50_ 0.66 μM/ml) showed potent HIV-1 RT inhibition, while compounds 4 and 5 exhibited low inhibition. Compounds 1 and 2 were also isolated from the hexane extract, however neither of these inhibited HIV-1 RT. As for the acetone fractions, fractions 1, 2 and 3 were evaluated for HIV-1 RT inhibition however, only fraction 2 presented a moderate activity where this fraction, which was analyzed by thin-layer chromatography (TLC), was suggested to contain cardenolides traces. Moreover, the methanol extract showed high HIV-1 RT inhibitory activity (83.3%), however, this value decreased to 38.6% after tannin removal. The ethyl acetate insoluble fraction from the methanol extract inhibited HIV-1 RT activity and presented low cytotoxicity, nevertheless, it was unable to inhibit IIIb/LAV replication. The *C. brasiliense* methanolic leaf extract showed inhibition of HIV-1 RT which may be attributed to the tannins present in the sample (Huerta-Reyes et al., 2004a) (Figure 3).

In another study by Zhou et al. (2000), isolation and characterization of eight new coumarins from *Ferula moschata* (H.Reinsch) Koso-Pol. the dry root methanolic extract and 19 other known coumarins were evaluated, as well as the anti-HIV activity and the inhibition of cytokine release of the extract. The author reports in his study that *F. moschata* is a rich source of coumarin derivatives. Compound 19 showed anti-HIV activity in that it inhibited HIV replication in H9 lymphocytes with EC50 values <0.10 mg/ml, in addition to inhibiting the growth of uninfected H9 cells with IC50 values > 100 mg/ml and a calculated therapeutic index (TI) > 1,000. In general, a TI > 5: 0 is considered to denote significant activity; compounds 11, 12, 13, 17 and 21 showed strong anti-HIV activity with TI values higher than 5.

## Clinical Studies

In a clinical study with 100 women, were randomly assigned to treatment with a Brazilian Peppertree (*Schinus terebinthifolius* Raddi-see Figure 2A) gel and with placebo. The results showed a cure rate of 84% in patients with symptomatic bacterial vaginosis treated with the Brazilian Peppertree gel, revealing a statistically significant difference compared to the cure rate found among patients receiving placebo (47.8%). The results, therefore, suggest the Brazilian Peppertree gel may be a safe and effective therapeutic alternative for bacterial vaginosis (Amorim and Santos, 2003). In another clinical study, 34 traditional Bapedi healers from the Limpopo Province in South Africa reported treatments for STDs during interviews, where *chlamydia* was one of the four common conditions stated. In terms of *chlamydia* treatment, eight plant species were identified: *Aloe marlothii* subsp. *marlothii*, *Eucomis pallidiflora* subsp. *pole-evansii*, *Gethyllis Namaquensis* (Schonland) Oberm., *Hypoxis obtusa* Burch. ex Ker Gawl., *Kleinia longiflora* DC., *Protea caffra* subsp. *caffra*, *Tribulus terrestris* L. and *Ziziphus mucronata* Willd (Figure 2A).

In a recently conducted clinical study with female patients who had vaginal trichomoniasis resistant to MTZ drugs it was found that patients who were also resistant to the MTZ and tinidazole combination received treatment with *Commiphora molmol* (Engl.) Engl. ex Tschirch, In a recently conducted clinical study with female patients who had trichomoniasis resistant to MTZ, MTZ and tinidazole combination, these received treatment with *Commiphora molmol* (Engl.) Engl. ex Tschirch. In addition, the *in vitro* effect against *T. vaginalis* of the *Punica granatum* L. pomegranate extract has been evaluated. The oil-resin myrrh-derived extract, *C. molmol* was administered to females resistant to MTZ and tinidazole in the form of two capsules (600 mg) six to eight days, 2 h before breakfast. All patients were seen immediately after completion of treatment and again 4–6 weeks later. In total, 33 cases were studied; 50% of patients (9/18) who received concomitant oral tinidazole were cured, but the cure rate was higher (73%) in patients (11/15) treated with a combination of oral MTZ and vaginal tinidazole.

At a pH of 4.65, *P. granatum* was effective against *T. vaginalis*, which was destroyed immediately at a concentration of 50–100 mg of extract and in half an hour at a concentration of 20 mg of extract. The author also reports that *P. granatum* extract with a concentration of 10%, demonstrated 100% effect against *T. vaginalis* in Diamond media (El-Sherbiny and El Sherbiny, 2011).

## Overall Conclusion and Future Perspectives

Complications caused by STDs are essential due to global public health problems, caused by social behaviour, vulnerability conditions that increase the risk of infection with various microorganisms and the risk of HIV infection. Natural bioactive compounds have positive effects against the viability of pathogenic microorganisms, compared to drugs used to treat STDs. They showed inhibitory effects on parasites, fungi, bacteria and viruses.

Future perspectives address the need for translational medicine studies with establishing exact therapeutic doses for humans. Coordinated multicenter clinical trials are also needed to accumulate larger, homogeneous cohorts in the selection of patients with STDs and the severity of their disease. Also, several clinical trials should use a randomized controlled design in order to obtain higher levels of evidence.

Limitations and clinical pitfalls in the clinical therapy of STDs using natural compounds derive from their bioavailability and low absorption, especially for topical application. Therefore, future research on pharmaceutical nanoformulations for ointments or gels as versatile drug delivery systems is needed. The use of drug vectors for the controlled release of bioactive natural compounds in a series of nanoformulations has multiple advantages: non-toxic, biodegradable, can incorporate both hydrophilic and lipophilic chemical compounds, can increase their circulation time, and can reach organs or tissues target, reduce the toxicity of the active substance and often improve its bioavailability. It would also be promising to conduct prospective studies using such bioactive compounds as adjuvants in the management of STDs, which would greatly shorten the production time of drugs with oral administration or topical application. Another limitation of our work derives from the fact that pharmacological evidence and molecular targets of action are gathered from recent meta-analyzes and not from personal data or individualized case presentations. But the included meta-analyzes contain a summary of the latest and most important studies on bioactive compounds effective in the pharmacotherapeutic management of STDs and as a result, based on scientific evidence this may be a strong point highlighting the effects of natural compounds as potential therapeutic agents.

Therefore, future efforts are needed for the generation of new drugs derived from natural compounds against STDs. To be used as safe complementary therapies in the management of STDs, more clinical trials are needed in the future.
